# Numerical investigation the hydrodynamic parameters of the flow in a wavy corrugated channel using different turbulence models

**DOI:** 10.1016/j.heliyon.2022.e11901

**Published:** 2022-11-30

**Authors:** Omid Ali Akbari, Hossein Haghjoo, Azher M. Abed, Mahsa Karimi, Ali Maghzian, Gholamreza Ahmadi Sheikh Shabani, Amirmasoud Anvari, Nevzat Akkurt, Davood Toghraie

**Affiliations:** a Department of Mechanical Engineering, Khomeinishahr Branch, Islamic Azad University, Khomeinishahr, Iran; b Air Conditioning and Refrigeration Techniques Engineering Department, Al-Mustaqbal University College, Babylon 51001, Iraq; c Mechanical Engineering Department, University of Kashan, Kashan, Iran; d Department of Renewable Energies and Environment, Faculty of New Science and Technologies, University of Tehran, Tehran, Iran; e Department of Mechanical Engineering, Iran university of science and technology, Tehran, Iran; f Munzur university, Department of Mechanical Engineering 62000 Tunceli, Turkey

**Keywords:** Turbulent flow, Numerical simulation, Pressure drop, Wavy channel, Turbulence models, Friction factor

## Abstract

In this research, turbulent flow numerical models in a wavy channel were investigated. The studied channel is simulated in two dimensions and symmetrically in the range of Reynolds numbers from Re=10,000 to 80,000. The significant cause of this research is to investigate and determine the appropriate method for estimating the behavior of turbulent flow in a wavy channel. In this research, the behavior of turbulent flow in a wavy channel will be simulated in 7 different ways, which are k-*ω* SST, k-*ϵ* RN, k-*ϵ* Realizable, k−*ϵ* Standard, k-*ω* Standard, Reynolds stress and Spalart-Allmaras. The findings of this research show that the impacts of the presence of flow viscosity (friction) and the presence of adverse pressure gradients are factors that strongly affect the velocity profiles in the upstream areas of the corrugated section. Among the studied models, due to better compatibility and guessing of flow and hydrodynamic properties, k-*ω* SST methods and Reynolds and Spalart-Allmaras stress are introduced as the best methods for such geometries. On the other hand, increasing the accuracy of other turbulence methods is related to the flow physics and geometric structure of each problem. In this research, the hydrodynamic parameters of the flow such as pressure drop, skin friction factor, and dynamic pressure drop coefficient and vortex contours, and pressure are plotted and described.

## 1 Introduction

Today, issues related to the field of fluid mechanics, and understanding its concepts in the fields of civil engineering, mechanical engineering, chemical engineering, and other engineering trends are very important, because each of them, according to the applications and needs, from the studies are used in the field of fluid mechanics [1], [2], [3], [4], [5], [6]. This need can be reduced to analytical and design issues, or to expand this branch of science, it can go beyond and achieve new achievements in this field [7], [8], [9], [10], [11], [12], [13], [14]. Turbulence study in the field of fluid mechanics constitutes a physical phenomenon of challenging issues and has a particular scientific value. In terms of engineering and application, the main problem is the prediction and inhibition of turbulence. The importance of this issue is compounded by the frequent presence of turbulent flows in nature and industry and their significant effects. Obviously, in most of the different applications of natural, technical, and environmental sciences, the occurrence of turbulence in flow and strengthening is our desired result. To apply and recognize turbulence, the predicted models for this phenomenon have been used by different researchers in different geometries. For instance, the Standard k-*ε* model [15] is one of the most common two-equation models in industrial applications due to its numerical stability and simplicity [16]. One of the main disadvantages of this model is that in areas with high curvature, the rate of turbulence energy generation is much higher than its actual value, which causes errors in calculating turbulence viscosity and related stresses [17]. Also, this model has low accuracy in simulating the boundary layer with an adverse pressure gradient [18]. However, the Realizable k-*ε* model has higher accuracy in rotational flows, boundary layers with adverse pressure gradient, and separation and eddy areas than other models of k-*ε* type [19]. The two-equation model SST k-*ω* is suitable for flow simulation in turbomachines because it has good accuracy in simulating areas with separation and adverse gradients [20]. In a three-dimensional numerical analysis using FLUENT software, Harikrishnan et al. [21] investigated the heat transfer parameters of a sinusoidal wavy channel with secondary waves. In this study, various parameters such as wave amplitudes, number of waves in the direction of flow, height and width of the channel, etc., were studied. This study investigates the effect of flow direction ripple for different Reynolds numbers in the range 2000 to 4000 and the secondary corrugated channel on the flow characteristics and heat transfer. Owing to the mixing of diverse flow directions, the heat transfer qualities of the secondary corrugated channel were determined to be greater. In the direction of flow, for instance, the temperature gradient in this channel is larger than in the corrugated channel. Sohankar et al. [22], at Re =1000, Pr=4.29, investigated computational simulations of heat transfer and pressure drop within three-row pipes as part of unique heat exchangers. Two pairs of winglet-type vortex generators (VGs) were positioned in the area of the first row and between the second and third rows in this investigation to boost the heat transfer rate. The impacts of geometric characteristics like longitudinal position and transverse angle of attack (VGs) were investigated. The findings reveal that, in the best scenario, the convective heat transfer coefficient and thermal performance coefficient improve by around 59 percent and 43 percent, respectively, when compared to geometry without (VG). The hydrothermal efficacy of corrugated or perforated fins (CPFs) as heat exchangers in a solar heating system was investigated in an experimental investigation by Khoshoghat et al. [23]. Moreover, using a combination of water and nanofluid as a fluid, this study studied parameters such as wave dimension ratio, nanofluid concentration, hole diameter, and flow velocity. CPFs had a greater heat transfer ratio and smaller pressure drop than standard examples, according to the findings. In addition, as compared to the base fluid, water and alumina nanoparticles had greater heat transfer coefficients and pressure drops. For tackling the issues of heat loss in electronic equipment, Wang et al. [24] carried out an experimental study. In a constant flux condition, the impacts of inlet temperature and structural characteristics like amplitude and wavelength on boiling heat transfer in semi-sinusoidal corrugated copper microchannels were investigated. Microchannels may be classified into four groups. Plain bottom microchannels have a higher heat flow and a lower pressure drop than half-corrugated microchannels. Furthermore, owing to the flow's boiling instabilities, the heat flux at 30°C intake temperature is larger than at the inlet temperature of 90°C, and all geometries have an identical function. Ganju et al. [25] examined turbulent channel flow in a fixed Reynolds number mode with numerical simulation. The flat and sinusoidal corrugated walls were compared. It was found that the wall waviness directly affects the internal flow region, and the outer region is ineffective. To study the influence of skewness on the flow heat transfer parameters, Harikrishnan et al. [26] undertook a three-dimensional computation in a corrugated channel. The skewness of the corrugated channel was discovered to generate a more powerful secondary flow, making the flow three-dimensional. A more powerful secondary flow, on the other hand, has an impact on the channel's thermo-hydraulic performance. Rashidi et al. [27] used thermohydraulic analysis and entropy generation to study turbulent flow in a corrugated channel. The goal of this research was to find the ideal settings for maximizing thermal performance while reducing irreversibility. The influence of different factors such as Reynolds number (Re), wave amplitude (a), and wavelength (k) on heat transfer, pressure drop, and entropy production was investigated as a result. The total thermal performance of the corrugated channel with a wave amplitude of 0.1 was considerably optimized for all Reynolds numbers, according to the findings. Alnak [28] used computational simulation employing the k−*ϵ* turbulence model to investigate heat transfer, pressure drop, and thermohydraulic efficiency in a triangular-section channel with rectangular baffles with different angles of settling. The Reynolds number studied in this study ranges from 1000 to 6000, and rectangular baffle settling angles of 30°, 45°, 60°, and 90° are considered. The Nusselt number for the corrugated channel with rectangular baffle and 90 ° angle of settling was determined to be 52.8% greater than 60° at Re=6000. Tokgoz et al. [29] studied flow hydrodynamics, turbulent flow properties, and time-dependent generated flow and vortices in a corrugated channel using a computer model. They looked at the impact of various phases, Re=3,000 to 6,000, and channel diameters. The largest local velocity value was discovered close to the wall. The thermal boundary layers in the corrugated channel are narrower than the thermal boundary layers in the straight channel flow. The energy is increased by the generation of vortices within the cavities. In Reynolds numbers, the usage of the corrugated channel results in a poorer thermal efficiency index than other Reynolds numbers. Kurtulmuşa et al. [30] investigated the factors that influence heat transfer in a corrugated channel. They provided the prior energy system's design specifications, practical constraints, and findings in a tabular format. Employing water/ZnO nanofluids, Kadhim et al. [31] investigated the flow structure and heat transfer characteristics within a corrugated curve channel computationally. Furthermore, utilizing the hydraulic performance technique, the existence of L-shaped baffles, the impact of waves, the configuration of baffles and their geometric elements, distinct corner angles, distinct blockage ratios in RE=8000-32000, and volume fraction of Zinc oxide nanoparticles in the range (SVF=0-4%) were investigated. For turbulence simulation, the - model was investigated. The findings revealed that heat transfer might be improved by the development of vortex flow and enhanced turbulence caused by corrugation and baffles. The linear baffle layout is also better than the staggered baffle design. Gao et al. [32] investigated the properties of three-dimensional turbulent flow and heat transfer in a channel with a corrugated wall and a constant temperature boundary condition computationally (the corrugated wall is sinusoidal in the stream-wise and span-wise directions). The wall is corrugated in the flow direction, sinusoidal, and flow width. The current shape enhances the pressure coefficient in the flat channel while decreasing the friction factor, according to the results. As a consequence of the increased pressure coefficient, the overall drag coefficient rises. With a blockage ratio of 0.25 and a corner angle of 30 °, the hydraulic, thermal performance (PEC) is about 1.99. However, many studies on the study of turbulence flow in different geometries were reviewed by researchers in recent decades. But there was a lack of a comprehensive study on the recognition and simulation of turbulent flow by various turbulence models. In this numerical study, the hydrodynamic parameters of the flow in a two-dimensional wavy channel are investigated and calculated using seven turbulence methods. The results of this research are plotted and described for parameters such as pressure drop, skin friction factor, and dynamic pressure drop coefficient and vortex contours, and pressure for the same conditions in the considered geometry.

## 2 Methodology

**Figure 1:**
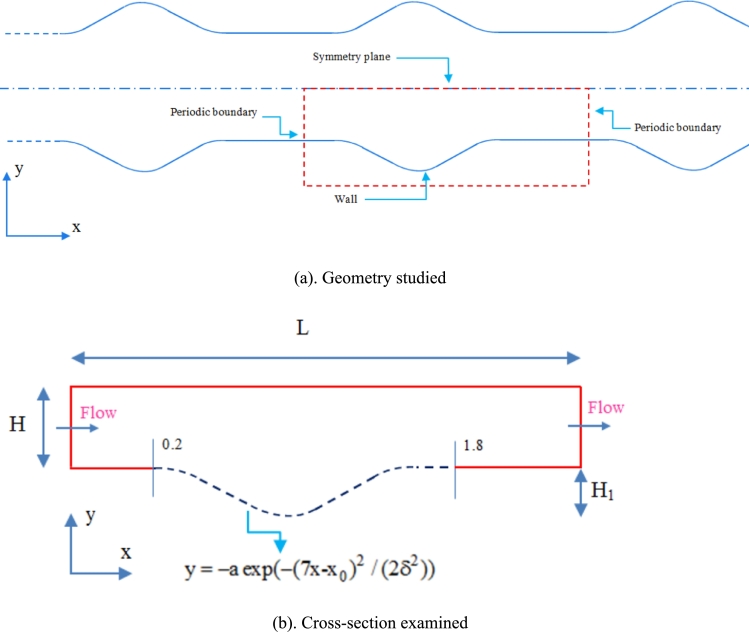
Schematic of the examined geometry.

In this research, the hydrodynamic behavior of turbulent flow in a wavy channel using 7 turbulence methods was studied. The geometry of the study is a wavy channel with a waveform with $y=-a\times \exp[-{\left(7x-x_{0}\right)}^{2}/(2\delta^{2})]$ function, the constants of which are $a=0.14,x_{0}=5.907,\delta =1.8219$. In this work, the effect of the wave of the wall on turbulent flow behavior by finite volume method in 2D space in this 2D channel is described. Fig. 1 describes the schematic of the studied geometry and the boundary conditions of the problem.

Table 1 describes and introduces the above geometry dimensions.

**Table 1:** Dimensions and geometry parameters introduced.

H	Height of channel	1
L	Channel pitch	3.68H
H_1_	Corrugation height	0.2H

The following values are used to nondimensionalize the parameters of this research *as Eq.* (1):

(1) $$Y=\frac{y}{H},\;X=\frac{x}{H},\;U=\frac{u}{u_{in}}$$

According to Fig. 1, the periodic condition is used in the input and output sections of the flow in the problem. The top wall of the channel also has a symmetry condition, which is drawn symmetrically to reduce the range and number of solution networks. In the lower corrugated wall, the condition of the wall is used and solved. In this issue, the boundary condition of non-slip on the walls is used. In general, this describes the hydrodynamics of the flow. This research is numerically simulated using the finite volume method in two-dimensional space. In this research, the above geometry will be investigated in 7 different ways of simulating turbulent flows, which are: k-*ω* SST, k-*ϵ* RN, k-*ϵ* Realizable, k−*ϵ* Standard, k-*ω* Standard, Reynolds stress, and Spalart-Allmaras.

## 3 The governing equations

The continuity equation is as Eq. (2):

(2) $$\frac{\partial \left(\bar{u_{i}}\right)}{\partial x_{i}}=0$$

The momentum equation is as Eq. (3):

(3) $$\frac{\partial }{\partial x_{j}}\left(\bar{u_{i}u_{i}}+\bar{u_{i}^{\prime }u_{j}^{\prime }}\right)=\frac{1}{\rho }\left(-\frac{\partial \bar{p}}{\partial x_{i}}+\frac{\partial \bar{\tau_{\mathrm{ij}}}}{\partial x_{i}}\right)$$

The scalar quantity equation is as Eq. (4):

(4) $$\frac{\partial }{\partial x_{j}}\left(\bar{u_{j}\emptyset }+\bar{u_{j}^{\prime }\emptyset^{\prime }}\right)=\frac{1}{\rho }\frac{\partial }{\partial x_{j}}\left(\Gamma \frac{\partial \bar{\emptyset }}{\partial x_{j}}\right)$$

which is the $\bar{\tau_{\mathrm{ij}}}$ components of the mean viscous stress tensor as Eq. (5):

(5) $$\bar{\tau_{\mathrm{ij}}}=\mu (\frac{\partial \bar{u_{i}}}{\partial x_{j}}+\frac{\partial \bar{u_{j}}}{\partial x_{i}})$$

These equations have the same structure as the original equations of mass continuity, momentum, and linear momentum equation, except that the new equations were for the intermediate variables, and the terms that express turbulence are added to these equations. The presence of turbulence terms, such as $\rho \bar{u_{i}^{\prime }u_{j}^{\prime }}$ (Reynolds stresses) and $\rho \bar{u_{i}^{\prime }\emptyset^{\prime }}$ (turbulent scalar flux) in the survival equations, means that these equations are not closed: That is, the number of variables is greater than the number of equations. To close these equations, the terms due to flow turbulence must be modeled in some way and lead to different turbulence models. The choice of model from the available models depends on the flow physics, accuracy, hardware features (CPU, RAM), and time required.

### 3.1 Two-equation model

The simplest and most complete turbulence models are the two-equation models. In this method, two distinct transfer formulas are used to independently display the length of the turbulence scale and the velocity of the turbulence scale. The most important difference between this model and other viscosity eddy is that two-equation models can be used to forecast turbulent flow characteristics without prior information on flow structure and geometry.

**Standard k-** *ε* **Model:**

The simultaneous solution yields the simultaneous solution of the two equations of turbulence kinetic energy k and energy dissipation rate *ε* (Eqs. (6) and (7))

(6) $$\frac{\partial }{\partial x_{i}}\left(ku_{i}\right)=\frac{1}{\rho }(\frac{\partial }{\partial x_{i}}\left\lfloor \left(\mu +\frac{\mu_{t}}{\sigma_{k}}\right)\frac{\partial k}{\partial x_{j}}\right\rfloor +G_{k}+G_{b}-\rho \varepsilon -Y_{M}+S_{k})$$

(7) $$\frac{\partial }{\partial x_{i}}\left(\varepsilon u_{i}\right)=\frac{1}{\rho }(\frac{\partial }{\partial x_{j}}\left\lfloor \left(\mu +\frac{\mu_{t}}{\sigma_{\varepsilon }}\right)\frac{\partial \varepsilon }{\partial x_{j}}\right\rfloor +C_{1\varepsilon }\frac{\varepsilon }{k}\left(P_{k}+C_{3\varepsilon }p_{b}\right)-C_{2\varepsilon }\rho \frac{\varepsilon^{2}}{k}+S_{\varepsilon })$$

In the above equations, $G_{k}$ produces kinetic energy from the mean velocity gradient and is obtained from *Eq.* ((8)).

(8) $$G_{k}=\frac{\partial u_{i}}{\partial x_{j}}\rho \bar{u_{i}^{\prime }u_{j}^{\prime }}$$

In the above equations $G_{b}$ is the kinetic energy production of the fluid buoyancy force, which is obtained from Eq. (9).

(9) $$G_{b}=\beta g_{i}\frac{\mu_{t}}{{\mathrm{pr}}_{t}}\frac{\partial T}{\partial x_{i}},\;{\mathrm{pr}}_{t}=\frac{1}{\alpha }=\frac{k}{\mu c_{p}}=0.85,\;\beta =-\frac{1}{\rho }{\left(\frac{\partial T}{\partial P}\right)}_{p},\;g=9.81m/s2$$

In the above equations $Y_{M}$ is the production of turbulence energy, and is obtained from the turbulence incompressible equations. A Speed of sound, $M_{t}$ is the turbulence Mach number (see Eq. (10)).

(10) $$Y_{M}=2\rho \varepsilon M_{t}^{2},\;M_{t}=\sqrt{\frac{k}{a^{2}}},\;a\equiv \sqrt{\gamma \mathrm{RT}}$$

In the above equations, $S_{k}$ and $S_{\varepsilon }$ are the energy source terms. $C_{2\varepsilon },C_{1\varepsilon }$ and $C_{3\varepsilon }$ are constant values and $\sigma_{k},\sigma_{\varepsilon }$ are Prandtl k and *ε*, respectively. $\mu_{t}$ is the turbulence viscosity which is obtained from Eq. (11) *and* Eq. (12)

(11) $$\mu_{t}=\rho c_{\mu }\frac{k^{2}}{\varepsilon }$$

(12) $$C_{1\varepsilon }=1.44,\;C_{2\varepsilon }=1.92,\;C_{3\varepsilon }=-0.33,\;\sigma_{k}=1.0,\;\sigma_{\varepsilon }=1.3,\;c_{\mu }=0.09$$

**RNG k-***ε* **Model**

The transfer equations for RNG k-*ε* are very identical to the standard k-*ε* equations. But with the slight differences as follows as Eqs. ((13) and (14)):

(13) $$\frac{\partial }{\partial x_{i}}(ku_{i})=\frac{1}{\rho }(\frac{\partial }{\partial x_{i}}(\alpha_{k}\mu_{\mathrm{eff}}\frac{\partial k}{\partial x_{i}})+G_{k}+G_{b}-\rho \varepsilon -Y_{M}+S_{k})$$

(14) $$\frac{\partial }{\partial x_{i}}(\rho \varepsilon u_{i})=\frac{1}{\rho }(\frac{\partial }{\partial x_{i}}(\alpha_{\varepsilon }\mu_{\mathrm{eff}}\frac{\partial \varepsilon }{\partial x_{j}})+C_{1\varepsilon }\frac{\varepsilon }{k}(G_{k}+G_{3\varepsilon }G_{b})-C_{2\varepsilon }\rho \frac{\varepsilon^{2}}{k}-R_{\varepsilon }+S_{\varepsilon })$$

The turbulent viscosity is derived from Eq. (15):

(15) $$\left(\frac{\rho^{2}K}{\sqrt{\varepsilon \mu }}\right)=1.72\frac{\overset{ˆ}{\nu }}{\sqrt{{\overset{ˆ}{\nu }}^{3}-1+C_{\nu }}}d\nu$$

where $C_{\nu }\approx 100\overset{ˆ}{\nu }=\frac{\mu_{\mathrm{eff}}}{\mu }$. It causes better modeling for low Reynolds flows and simulations on the side of the wall. The inverse Prandtl number $\alpha_{k}$ and $\alpha_{\varepsilon }$ are obtained by Eq. (16):

(16) $${\left|\frac{\alpha -1.3929}{\alpha_{0}-1.3929}\right|}^{0.6321}{\left|\frac{\alpha +2.3929}{\alpha_{0}+2.3929}\right|}^{0.3679}=\frac{\mu_{\mathrm{mol}}}{\mu_{\mathrm{eff}}}$$

where $\alpha_{0}=1$ and for high Reynolds is $\alpha_{k}=\alpha_{\varepsilon }\approx 1.39\frac{\mu_{\mathrm{mol}}}{\mu_{\mathrm{eff}}}\ll 1$. The fundamental difference between the k-*ε* RNG model and the standard k-*ε* model is the additional expression $R_{\varepsilon }$ in equation *ε*, which is obtained according to Eq. (17):

(17) $$R_{\varepsilon }=\frac{C_{\mu }\rho \eta^{3}\left(1-\eta /\eta_{0}\right)}{\left(1+\beta \eta^{3}\right)K}$$

where:

$$C_{2\varepsilon }=1.68,\;C_{\mu }=0.0845,\;\beta =0.012,\;\eta =SK/\varepsilon ,\;\eta_{0}=4.38,\;C_{1\varepsilon }=1.42$$

**Realizable k-***ε* **Model**

The realizable model is a relatively new model that is described as Eqs. ((18) to (33)):

(18) $$\frac{\partial }{\partial x_{i}}(ku_{i})=\frac{1}{\rho }(\frac{\partial }{\partial x_{i}}\lfloor (\mu +\frac{\mu_{t}}{\sigma_{k}})\frac{\partial k}{\partial x_{j}}\rfloor +G_{k}+G_{b}-\rho \varepsilon -Y_{M}+S_{k})$$

(19) $$\frac{\partial }{\partial x_{i}}(\varepsilon u_{i})=\frac{1}{\rho }(\frac{\partial }{\partial x_{j}}\lfloor (\mu +\frac{\mu_{t}}{\sigma_{\varepsilon }})\frac{\partial \varepsilon }{\partial x_{j}}\rfloor +\rho C_{1}S\varepsilon -\rho C_{2}\frac{\varepsilon }{K+\sqrt{\nu \varepsilon }}+C_{1\varepsilon }\frac{\varepsilon }{K}C_{3\varepsilon }G_{b}+S_{\varepsilon })$$

(20) $$\mu_{t}=\rho c_{\mu }\frac{k^{2}}{\varepsilon }$$

(21) $$c_{\mu }=\frac{1}{A_{0}+A_{s}\frac{U^{⁎}k}{\varepsilon }}$$

(22) $$U^{⁎}=\sqrt{S_{\mathrm{ij}}S_{\mathrm{ij}}+{\overset{ˆ}{\Omega }}_{\mathrm{ij}}{\overset{ˆ}{\Omega }}_{\mathrm{ij}}}$$

(23) $${\overset{ˆ}{\Omega }}_{\mathrm{ij}}=\Omega_{\mathrm{ij}}-2\varepsilon_{\mathrm{ijk}}\omega_{k}$$

(24) $$\Omega_{\mathrm{ij}}=\bar{\Omega_{\mathrm{ij}}}-\varepsilon_{\mathrm{ijk}}\omega_{k}$$

(25) $$A_{s}=\sqrt{6}\cos\emptyset$$

(26) $$\emptyset =\frac{1}{3}\cos^{-1}(\sqrt{6}w)$$

(27) $$w=\frac{S_{\mathrm{ij}}S_{\mathrm{jk}}S_{\mathrm{ki}}}{{\overset{ˆ}{S}}^{3}}$$

(28) $$\overset{ˆ}{S}=\sqrt{S_{\mathrm{ij}}S_{\mathrm{ij}}}$$

(29) $$S_{\mathrm{ij}}=\frac{1}{2}(\frac{\partial U_{j}}{\partial {\mathrm{Ux}}_{i}}+\frac{\partial U_{i}}{\partial {\mathrm{Ux}}_{j}})$$

(30) $$C_{1}=\max[0.43,\frac{\eta }{\eta +5}]$$

(31) η=SK/ε

(32) $$S=\sqrt{2S_{\mathrm{ij}}S_{\mathrm{ij}}}$$

Constant values are:

(33) $$C_{1\varepsilon }=1.44,\;C_{2}=1.9,\;\sigma_{k}=1.0,\;\sigma_{\varepsilon }=1.2,\;A_{0}=4.04$$

**Standard k-***ω* **model**

The standard k-*ω* scheme is an experimental model centered on experimental transfer formulas for k and *ω*. These equations can be expressed for k and *ε*. The transfer equations for k and *ω* are as *Eqs*. ((34) *and* (35)):

(34) $$\frac{\partial }{\partial x_{i}}(Ku_{i})=\frac{1}{\rho }(\frac{\partial }{\partial x_{i}}(\Gamma_{k}\frac{\partial k}{\partial x_{i}})+G_{k}-Y_{k}+S_{k})$$

(35) $$\frac{\partial }{\partial x_{i}}(\omega u_{i})=\frac{1}{\rho }(\frac{\partial }{\partial x_{j}}(\Gamma_{\omega }\frac{\partial \omega }{\partial x_{j}})+G_{\omega }-Y_{\omega }+S_{\omega })$$

In the above equations, $G_{k}$ is the kinetic energy production rate from the mean velocity gradient and $G_{\omega }$ is the *ω* production rate from the flow. $\Gamma_{k}$ and $\Gamma_{\omega }$ indicate the effective diffusion of k and *ω*. $Y_{k}$ and $Y_{\omega }$ are the diffusion rates k and *ω* due to turbulence. $S_{k}$ and $S_{\omega }$ are also source values obtained from *Eqs*. ((36) *and* (37)).

(36) $$\Gamma_{k}=\mu +\frac{\mu_{t}}{\sigma_{k}}$$

(37) $$\Gamma_{\omega }=\mu +\frac{\mu_{t}}{\sigma_{\omega }}$$

$\sigma_{k}$ and $\sigma_{\omega }$ are Prandtl numbers for k and *ω*. The turbulent viscosity ($\mu_{t}$) is obtained using k and *ω* as *Eq.* (38):

(38) $$\mu_{t}=\alpha^{⁎}\frac{\rho k}{\omega }$$

The coefficient $\alpha^{⁎}$ viscosity diffusion in flows which Reynolds is low, which is obtained as *Eq.* (39):

(39) α⁎=α∞⁎(α0⁎+Ret/Rk1+Ret/Rk)

where Ret=ρkμω, $R_{k}=6$, $\alpha_{0}^{⁎}=\frac{\beta_{i}}{3}$, $\beta_{i}=0.072$. In high Reynolds numbers $\alpha^{⁎}=\alpha_{\infty }^{⁎}=1$ term $G_{k}$, the kinetic energy output is obtained from *Eq.* (40).

(40) $$G_{k}=\frac{\partial u_{j}}{\partial x_{j}}\rho \bar{u_{i}^{\prime }u_{j}^{\prime }}$$

Using the Boussinesq hypothesis, this relationship can be written as *Eq.* (41):

(41) $$G_{k}=\mu_{t}S^{2}$$

where S is the modulus of the strain tensor rate, which is described as the k-*ε* model. The production rate *ω* is indicated by *Eq.* (42):

(42) $$G_{\omega }=\alpha \frac{\omega }{k}G_{k}$$

$G_{k}$ is obtained from Eq. (41) and the coefficient *α* is obtained by *Eq.* (43).

(43) α=α∞α⁎(α0⁎+Ret/Rω1+Ret/Rω)

here $R_{w}=2.95$ and α⁎.Ret=ρkμω and in the above Reynolds is $\alpha =\alpha_{\infty }=\;1$ and the loss rate k is obtained by *Eq.* (44):

(44) $$Y_{k}=\rho \beta^{⁎}f_{\beta^{⁎}}k\omega$$

where $f_{\beta^{⁎}}$ is obtained from Eq. (45):

(45) $$f_{\beta^{⁎}}=\left\{ \begin{array}{cc} 1,\; & x_{k}\leq 0\\ \frac{1+680x_{k}^{2}}{1+400x_{k}^{2}},\; & x_{k}>0 \end{array}\right.$$

Also see Eqs. ((46) *to* (57)):

(46) $$x_{k}\equiv \frac{1}{\omega^{3}}\frac{\partial k}{\partial x_{j}}\frac{\partial \omega }{\partial x_{j}}$$

(47) βi⁎=β∞⁎(4/15+(Ret/Rβ)41+(Ret/Rβ)4)

(48) $$\zeta^{⁎}=1.5,\;R_{\beta }=8,\;\beta_{\infty }^{⁎}=0.09$$

(49) $$Y_{\omega }=\rho \beta f_{\beta }\omega^{2}$$

(50) $$f_{\beta }=\frac{1+70x_{\omega }}{1+80x_{\omega }}$$

(51) $$x_{\omega }=\left|\frac{\Omega_{\mathrm{ij}}\Omega_{\mathrm{jk}}S_{\mathrm{ki}}}{{\left(\beta_{\infty }^{⁎}\omega \right)}^{3}}\right|$$

(52) $$\Omega_{\mathrm{ij}}=\frac{1}{2}\left(\frac{\partial u_{i}}{\partial x_{j}}-\frac{\partial u_{j}}{\partial x_{i}}\right)$$

(53) $$\beta =\beta_{i}\left[1-\frac{\beta_{i}^{⁎}}{\beta_{i}}\zeta^{⁎}F\left(M_{t}\right)\right]$$

(54) $$F\left(M_{t}\right)=\left\{ \begin{array}{cc} 0,\; & M_{t}\leq M_{t0}\\ M_{t}^{2}-M_{t0}^{2},\; & M_{t}>M_{t0} \end{array}\right.$$

(55) $$M_{t}^{2}=\frac{2k}{a^{2}}$$

(56) $$M_{t0}=0.25$$

(57) $$a=\sqrt{\gamma \mathrm{RT}}$$

In high Reynolds flows $\beta_{i}^{⁎}=\beta_{\infty }^{⁎}$ and for incompressible flow $\beta^{⁎}=\beta_{i}^{⁎}$, also constant values are obtained from Eqs. (58) to (59):

(58) $$\alpha_{\infty }^{⁎}=1,\;\alpha_{\infty }=0.52,\;\alpha_{0}=\frac{1}{9},\;\beta_{\infty }^{⁎}=0.09,\;\beta_{i}=0.072,\;R_{\beta }=8$$

(59) $$R_{k}=6,\;R_{\omega }=2.95,\;\zeta^{⁎}=1.5,\;M_{t0}=0.25,\;\sigma_{k}=2.0,\;\sigma_{\omega }=2.0$$

**SST k-***ω* **model**

This model works well for areas near and far from the wall and can be used for low and high Reynolds numbers. This model is presented by Eqs. (60) to (61):

(60) $$\frac{\partial }{\partial x_{i}}(ku_{i})=\frac{1}{\rho }(\frac{\partial }{\partial x_{j}}(\Gamma_{k}\frac{\partial k}{\partial x_{j}})+\overset{ˆ}{G_{k}}-Y_{k}+S_{k})$$

(61) $$\frac{\partial }{\partial x_{j}}(\rho \omega u_{j})=\frac{1}{\rho }(\frac{\partial }{\partial x_{j}}(\Gamma_{\omega }\frac{\partial \omega }{\partial x_{j}})+G_{\omega }-Y_{\omega }+D_{\omega }+S_{\omega })$$

In the above equations, $\overset{ˆ}{G_{k}}$ kinetic energy production rates are obtained from the mean velocity gradient and $G_{\omega }$ production rates of *ω* which are calculated from the mean flow. $\Gamma_{k}$ and $\Gamma_{\omega }$ indicate the effective diffusion of k and *ω*. $Y_{k}$ and $Y_{\omega }$ are the loss rates k and *ω* due to turbulence. $S_{k}$ and $S_{\omega }$ are also source values.

## 4 Grid independency and solution assumptions

**Figure 2:**
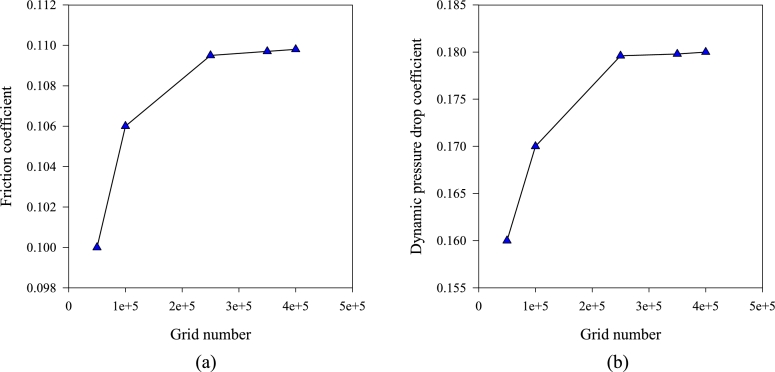
Graph of changes in friction factor and dynamic pressure drop coefficient per number of different grids.

Because the type of grid and its number can have a great impact on the answers and the method of the numerical solution and the resulting error, for this reason, in this study, an attempt was made to use a structured multi-book grid. In the present study, five grids from $50\times 10^{3}$ to $400\times 10^{3}$ grids were used to solve grid independence. Problem-solving was very sensitive to the number of grid points and there are small vortices near solid walls. Therefore, choosing the number of grids is very important. It should be noted that for a grid with fewer points and more than the above range, physically acceptable results have not been achieved. Due to the high pressure near the gradient channel wall, the size of the grid near these walls becomes too small, and the boundary layer grid will be used. Fig. 2 shows the diagrams of friction factor and dynamic pressure drop for five different grid numbers. It is observed that the changes of the above parameters for the number of $250\times 10^{3}$ grid number does not change much compared to the higher number of grid and if the $400\times 10^{3}$ grid number is used; the computational time will increase. Therefore, due to the high accuracy and low computational time required, the grid $250\times 10^{3}$ was selected for all calculations.

## 5 Discussion and results

### 5.1 Validation

**Figure 3:**
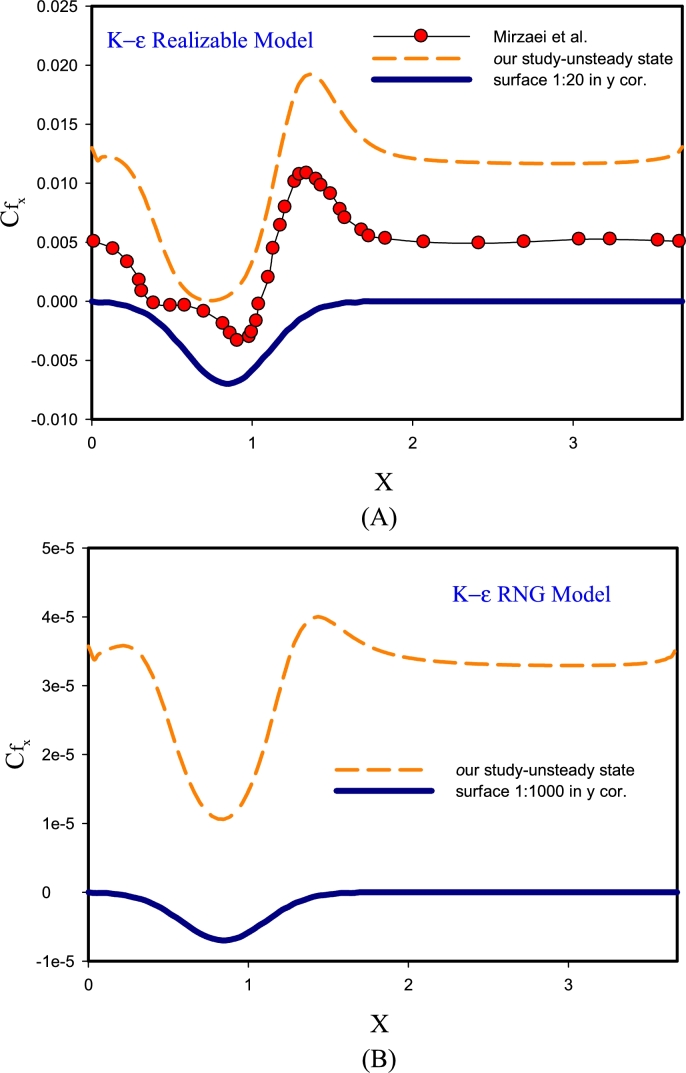
Validation of results for Re= 10000 by solving DNS in unsteady-state for k-*ϵ* RNG, k-*ϵ* Realizable models.

Fig. 3 displays the validation of the findings for Re=10000 with DNS solution [19] for k-*ϵ* RNG, k-*ϵ* Realizable models. According to Fig. 3-(A), using the k-*ϵ* Realizable method can have compatible solutions with precise DNS solutions [33]. Of course, the amount of error is also quite obvious in Figure A diagram. According to the diagram, it can be said that the k-*ϵ* Realizable method in estimating the flow separation region could not guess the flow behavior well. However, in terms of changes after the corrugated region and the reattachment region, the behavior of this method in guessing the local friction factor parameter has had a good trend. The reason for this behavior can be because this method predicts the flow propagation rate in jets and fountains in a better plane and circular and this geometry is inherently different. In Figure B, the behavior of the friction factor is predicted using the high-error k-*ϵ* RNG method. On average, this method predicts the flow behavior about 1.5 times less and the location of separation and vortex is not considered correctly. It exists to determine the viscosity of the flow and is strongly dependent on the turbulence of the flow. This may be due to differences in flow dependence and its effect on that function.

**Figure 4:**
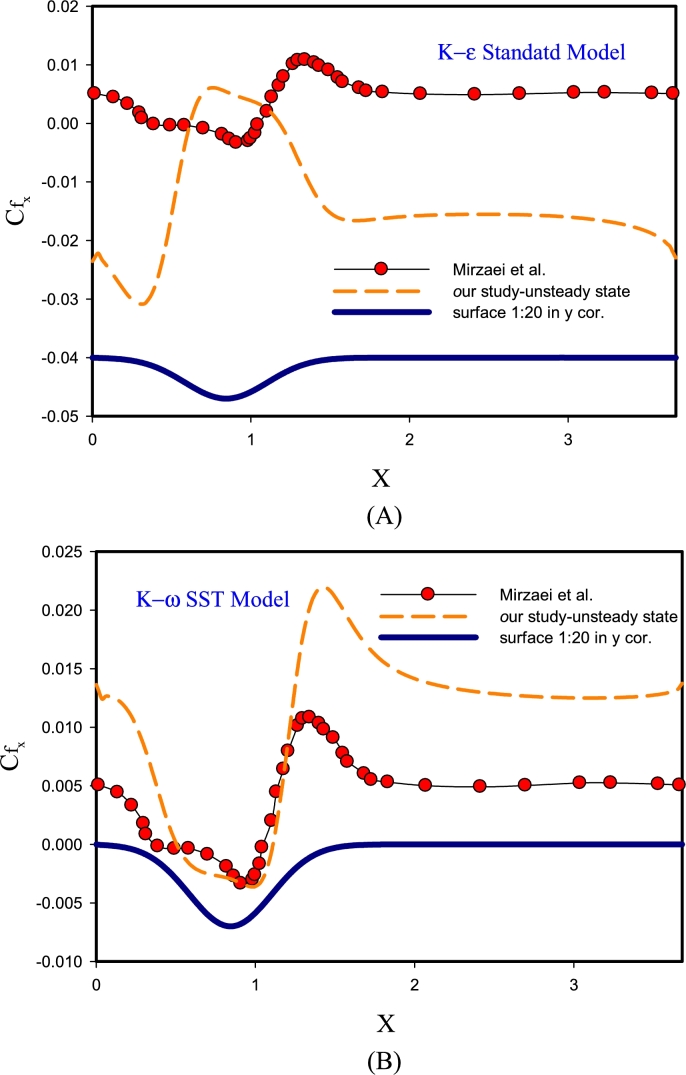
Validation of results for Re= 10000 by solving DNS in unsteady-state for models from k-*ω* SST, k−*ϵ* Standard.

Fig. 4 presents the validation of the results for the Re=10000 by solving the unsteady-state DNS for k-*ω* SST, and k−*ϵ* Standard models. Fig. 4-(A) compares the changes in the local friction factor on the corrugated wall for the Standard k−*ϵ* turbulence method and the DNS reference solution. In this model, guessing the friction factor for this geometry is not done correctly. The reason for this behavior is that this method was one of the first methods to determine turbulent behavior, and in this two-equation model, its equations are quasi-experimental with experimental observations and phenomenological considerations, which this behavior has erred in the results of this method, and because of this behavior, other methods of k−*ϵ* RNG, k−*ϵ* Realizable models were created to cover the error of this method. In Fig. 4-(B) the determination of the friction factor behavior with the DNS reference is compared with the k−*ϵ* SST model. According to this graph, it can be seen that this model predicts the friction factor behavior with appropriate accuracy, especially in the area of separation and reattachment of the flow. In this model, k−*ϵ* SST has the function of defining the Blending Function. And after deriving from the k−*ϵ* and k-*ω* models, it predicts the results of the flow behavior in the upper Reynolds areas (center of the channel) and the lower Reynolds areas (areas close to the wall), respectively, quite well.

**Figure 5:**
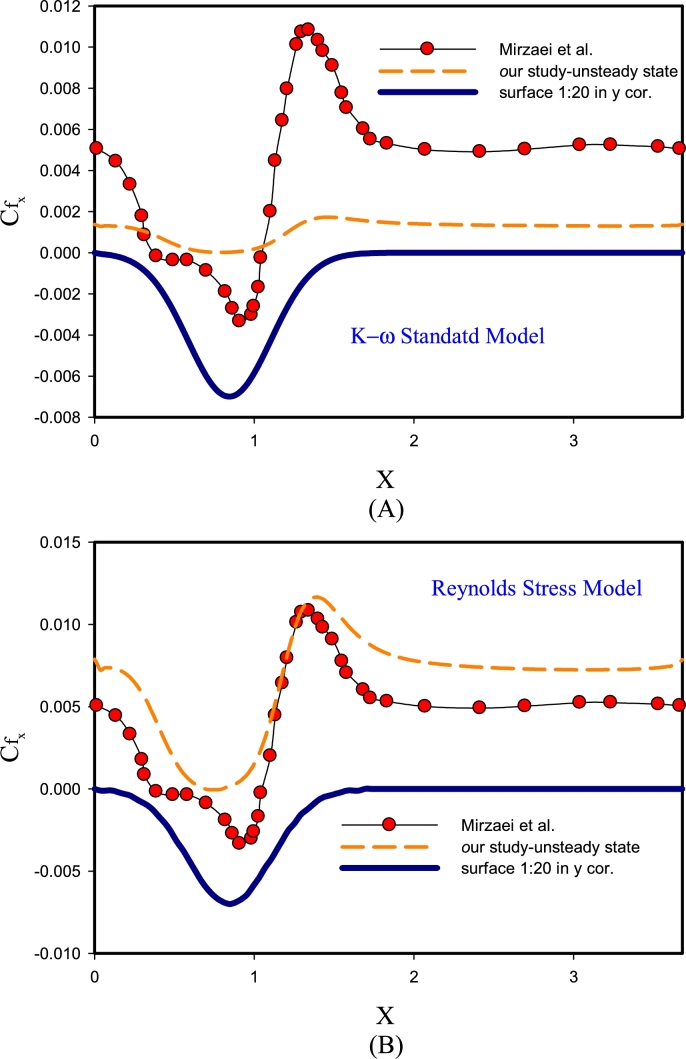
Validation of results for Re=10000 by solving DNS in unsteady-state for k−*ϵ* Standard, Reynolds stress models.

In Fig. 5, validation of the results of the behavior of the local friction factor on the corrugated wall for Re=10000 by solving the DNS in the unsteady state is presented for the standard k−*ϵ* Reynolds stress models. Fig. 5-(A) shows the k−*ϵ* Standard turbulence model, in which the guessing of the friction factor is inappropriate and with a high error. The reason for this behavior is due to better determination, estimation, and guessing of this flow for mixed layer flows, distant vortices, and circular and flat plate jets and free shear flow, and is used for finite flows to the wall in a limited way. Fig. 5-(B) shows the Reynolds stress turbulence model, in which the friction factor is estimated with a small error. The reason for this behavior is that in this method, compared to two-equation and single-equation models, another equation is used. In general, for two-dimensional flows, this method simulates with 5 equations and in three-dimensional mode with 7 equations. According to the shape of the Reynolds stress model, both in the separation and flow reattachment point, the estimation and guessing of the behavior is presented with a suitable error.

**Figure 6:**
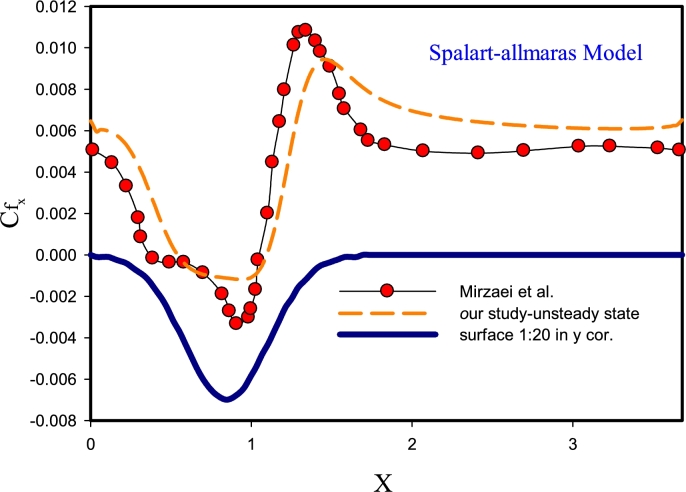
Validation of results for Re=10000 by solving DNS in the unsteady-state for Spalart-Allmaras model.

In Fig. 6, validation of the results for Re=10000 is performed by solving the unsteady-state DNS for the Spalart-Allmaras model. The Spalart-Allmaras method is a relatively simple single-equation model, in which an equation for turbulent viscosity is solved, and it does not need to calculate the characteristic length depending on the thickness of the shear layer. This method is suitable for wall flows and boundary layers with an adverse pressure gradient, and this model uses wall functions and this method has made this method the best option for approximate and initial solutions on large cells. The sensitivity of the mesh (distortion) near the wall is less felt in this method. The above characteristics have caused the Spalart-Allmaras method to perform behavior guessing with great accuracy among the studied methods.

### 5.2 Investigation of friction factor and dynamic pressure drop and average pressure drop

Table 2, Table 3, Table 4 show the characteristics of the average friction factor on the corrugated wall, the static pressure drop in the centerline, and the dynamic pressure drop coefficient on the corrugated wall, respectively. In this study, different values of flow parameters are reported in Re= 10000, 40000, and 80000. In many models, the results are well-matched and in others, the results are not reliable.

**Table 2:** Mean values of the friction factor on the corrugated wall of the channel.

Re	k-*ϵ* Realizable	k-*ϵ* RNG	k−*ϵ* Standard	k-*ω* SST	k-*ω* Standard	Reynolds stress	Spalart-Allmaras	
10000	0.01072	0.0216	−0.0176	0.011233	0.00089	0.00646	0.005179	Cf
40000	0.2657	0.24153	26.7229	0.10063	0.2225	0.15448	0.06524	
90000	0.965087	0.31345	3.7866	0.1095	1.9355	0.61084	0.19705

**Table 3:** Medium pressure drop values on the central stream line.

Re	k-*ϵ* Realizable	k-*ϵ* RNG	k−*ϵ* Standard	k-*ω* SST	k-*ω* Standard	Reynolds stress	Spalart-Allmaras	
10000	0.4669	0.0491	0.72533	0.734151	0.01869	0.180512	0.27346	P (pa)
40000	30.7132	36.4693	7474.283	13.4231	38.5676	14.05162	8.5601	
90000	157.2811	49.884	816.664	15.0096	659.37	88.5816	38.8118

**Table 4:** Mean values of dynamic pressure drop coefficient on the corrugated channel wall.

Re	k-*ϵ* Realizable	k-*ϵ* RNG	k−*ϵ* Standard	k-*ω* SST	k-*ω* Standard	Reynolds stress	Spalart-Allmaras	
10000	0.020971	0.01312	0.030747	0.00881	0.000227	0.009563	0.01241	Cp
40000	0.08646	1.6396	339	0.16058	0.4619	0.62069	0.3815	
90000	0.44386	2.2447	37.027	0.1796	7.971	3.7708	1.8607	

### 5.3 Axial velocity changes

**Figure 7:**
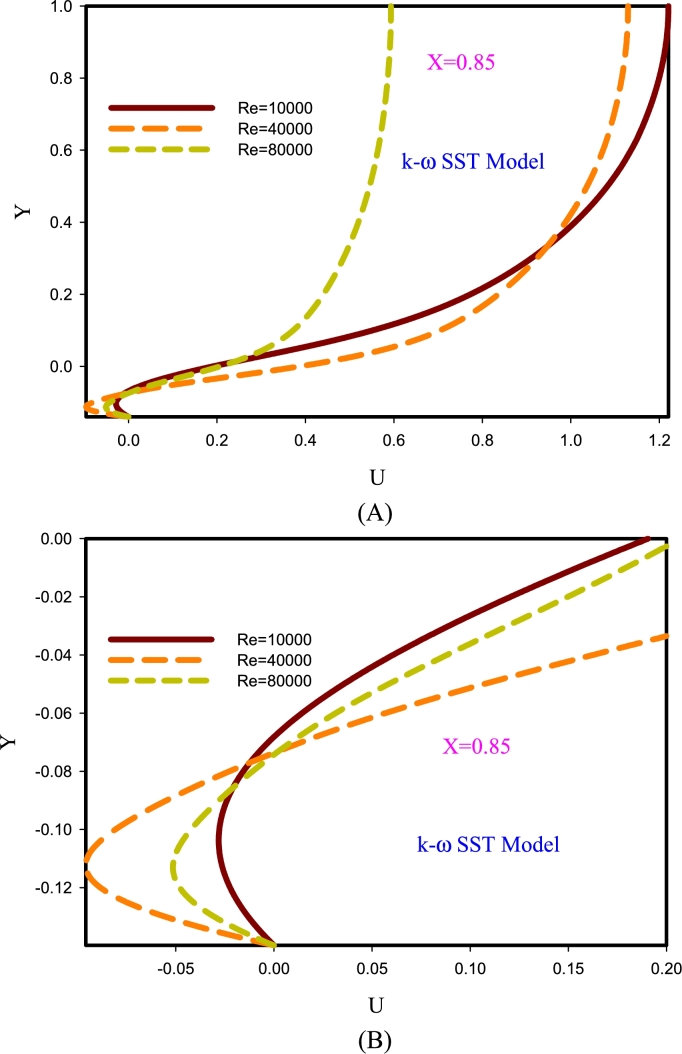
Diagrams of axial velocity changes in section X = 0.85.

Fig. 7 describes the diagrams of axial velocity changes in the model k-*ω* SST at section X=0.85. This section is located in the wavy area. The purpose of this study is to describe the flow behavior of different Reynolds numbers in the corrugated region. As the fluid reaches the corrugated area due to changes in fluid velocity components, the axial velocity is also affected. Due to the separation of the current due to the pressure gradient, the adverse pressure is reversed in the dimple region. The layers above the surface pass over the dimple due to the accompanying fluid flow, and because at this point the level has increased and the velocity has decreased. Hence charts usually have less than one range. The effects of flow viscosity (friction) and the presence of adverse pressure gradients are factors together, and the velocity profiles in the upstream areas of the corrugated section are strongly affected. The decrease in local velocity among the graphs at Re=10000 has the greatest effect.

**Figure 8:**
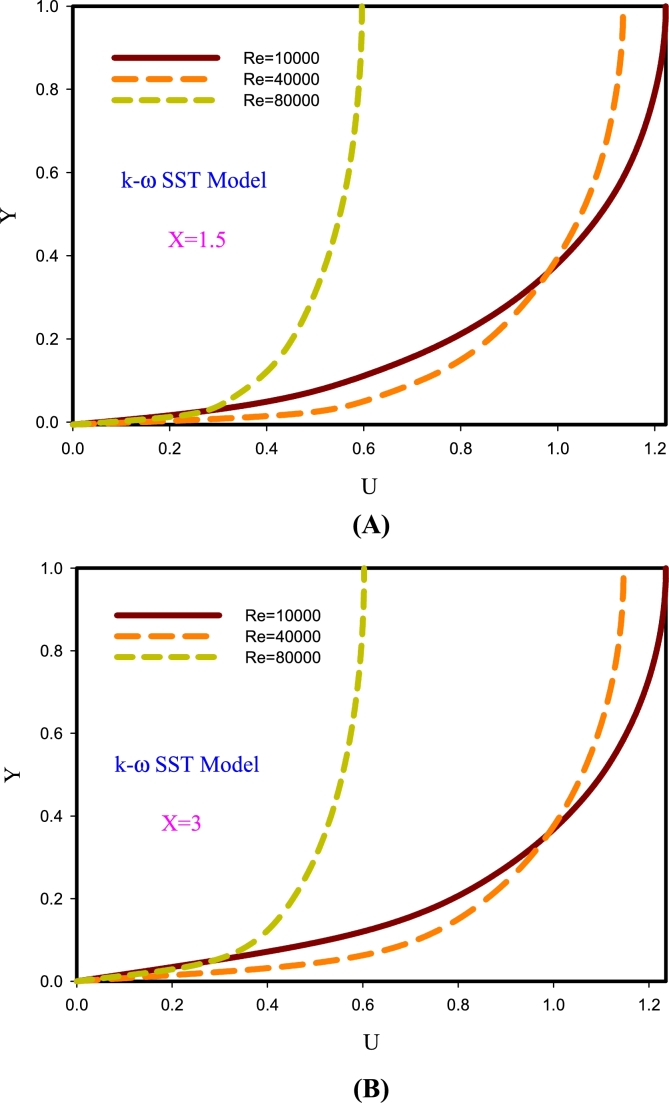
Diagrams of axial velocity changes in section X = 0.85.

Fig. 8 describes the diagrams of axial velocity and shows changes in section X=1.5 and X=3. According to the graphs, the rate of deceleration of velocity in sections X=1.5 (Fig. 8A) and X=3 (Fig. 8B) in Re=80000 is less than the Re=40000 and 10000. The cause of this behavior is due to the higher momentum of fluids at higher velocities, and at higher Reynolds numbers the effects of surface diffusion to higher fluid layers are less.

### 5.4 Static pressure contours

**Figure 9:**
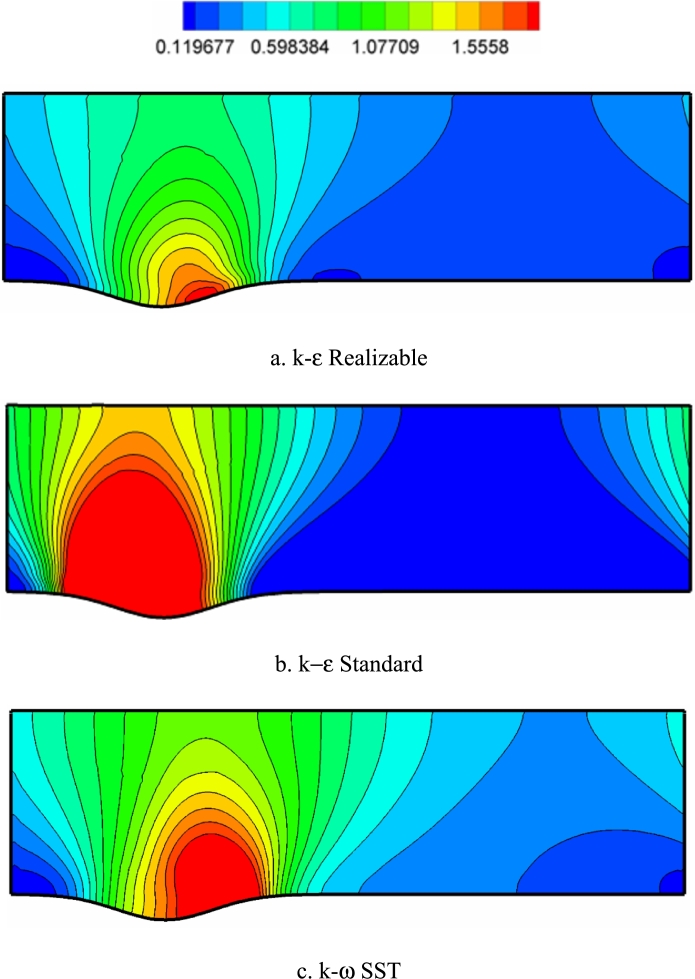
Pressure distribution contours (with pascal unit) in Re=1000.

**Figure 10:**
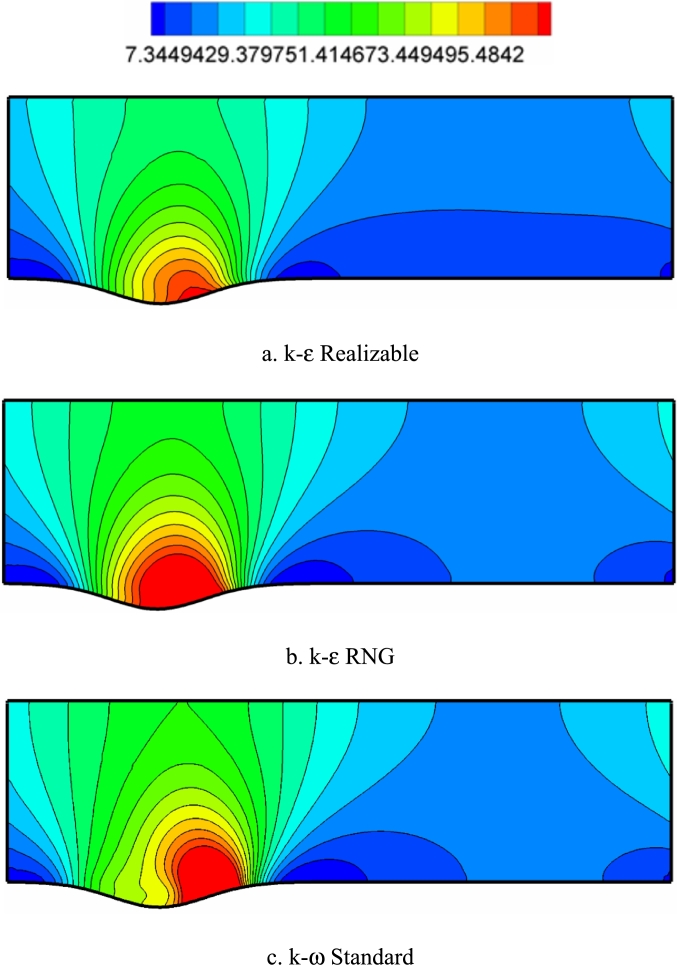
Pressure distribution contours (with pascal unit) in Re=40,000.

**Figure 11:**
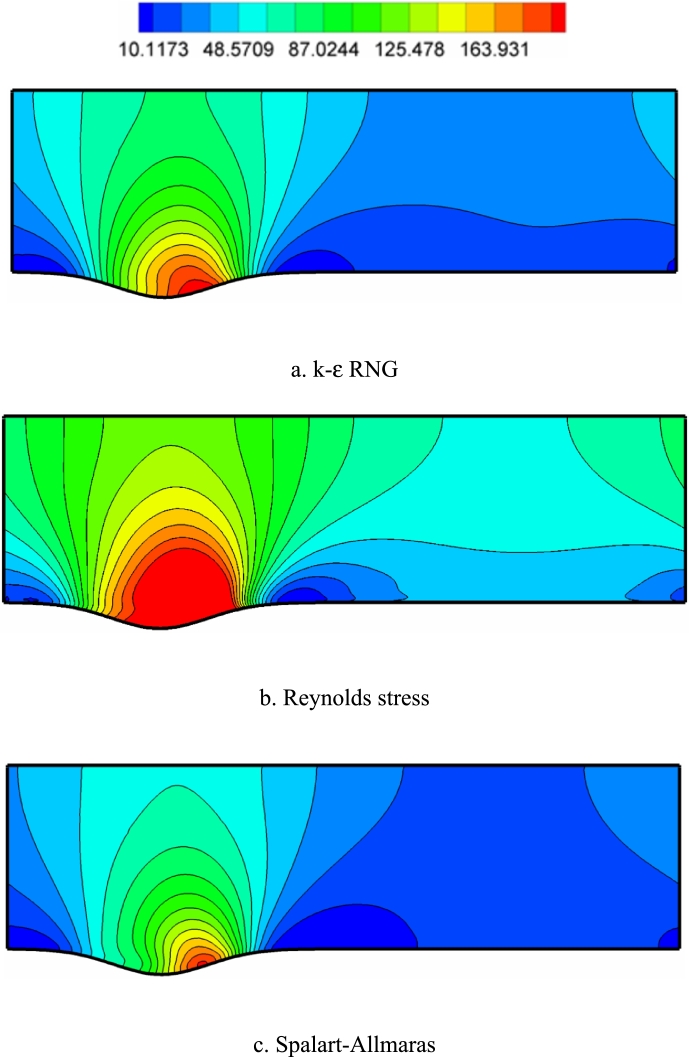
Pressure distribution contours (with pascal unit) in Re=80,000.

Figure 9, Figure 10, Figure 11 show the static pressure contours for Re=10000, 40000, and 80000, respectively. With the entry of fluid into the cross-section of the channel due to the presence of solid walls and the presence of geometric factors such as the presence of a wave at the bottom of the channel, the amount of velocity field is affected and the pressure drop increases in proportion to its changes. Among all the graphs, the highest pressure drop occurred in the corrugated section, which is due to the inverse ratio of velocity and pressure in ultrasonic flows. Also, the cross-section with the highest static pressure increase is the forehead of the surface collision. After the fluid passes through the corrugated section, due to the uniformity of the channel section (re-narrowing), the amount of momentum of the fluid increases, and the pressure drop is less.

### 5.5 Vorticity contours

**Figure 12:**
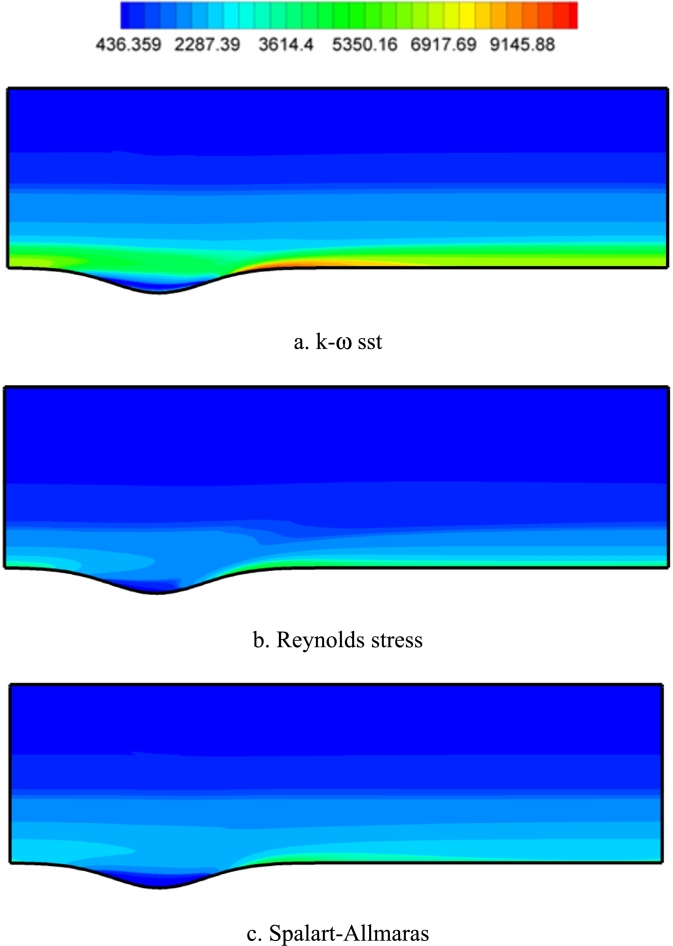
Vorticity contours (with s^−1^ unit) in Re=10000.

**Figure 13:**
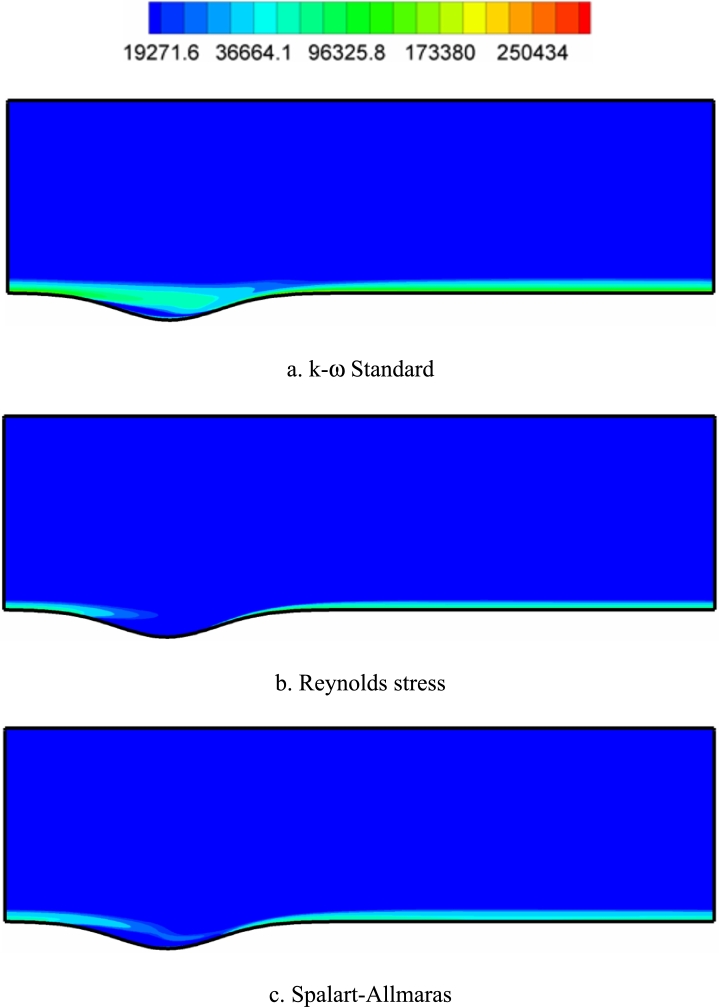
Vorticity contours (with s^−1^ unit) in Re=40,000.

**Figure 14:**
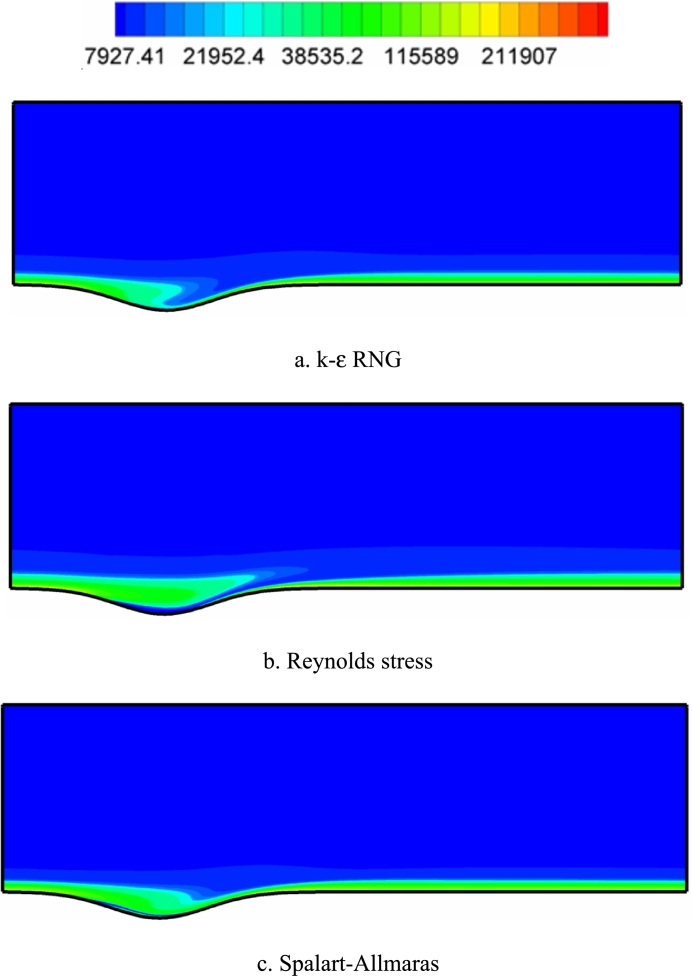
Vorticity contours (with s^−1^ unit) in Re=80,000.

Figure 12, Figure 13, Figure 14 show the contours of the vorticity value in the Re=10000, 40000, and 80000. Any factor in the flow motion that prevents the stream line from moving directly can produce vorticity. In all contours, the most vorticity of production are the areas close to the solid boundary and the areas before and after the corrugated area of the channel. Also, increasing the velocity of the fluid (Re) increases the vorticity. In all diagrams, as the fluid velocity increases, the vorticity change to the corrugated area of the channel. The presence of adverse gradients due to surface changes also enhances vorticity. In many turbulence models, the behavior determination of the vortex value is the same. The determination of behavior depends on the accuracy of the type of turbulence model and its specific application.

### 5.6 Turbulence intensity

**Figure 15:**
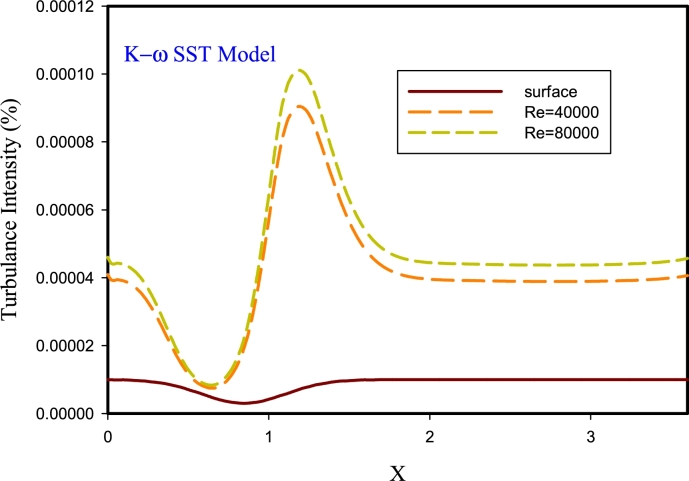
Turbulence intensity diagram for k-*ω* SST model at Re=40000 and 80000.

In Fig. 15, a turbulence intensity diagram for different models at Re=40,000 and 80,000 is plotted on the corrugated wall in the k-*ω* SST turbulence method. According to the graph, the intensity of turbulence depends on the Reynolds number. As the fluid velocity increases, the amount of velocity components increases, and, for the same reason, the turbulence intensity increases. In the path of fluid movement, the velocity decreases as the fluid enters the corrugated area and the channel opens. Hence, the number of fluid momentum decreases, and consequently the turbulence intensity decreases. Also, when the fluid hits the forehead of the corrugated area, the intensity of turbulence increases, and turbulence in these areas becomes important. After the fluid passes through the corrugated section, the turbulence intensity decreases due to the re-separation of the flow until the fluid hits the surface.

### 5.7 Turbulence viscosity and axial velocity counters

**Figure 16:**
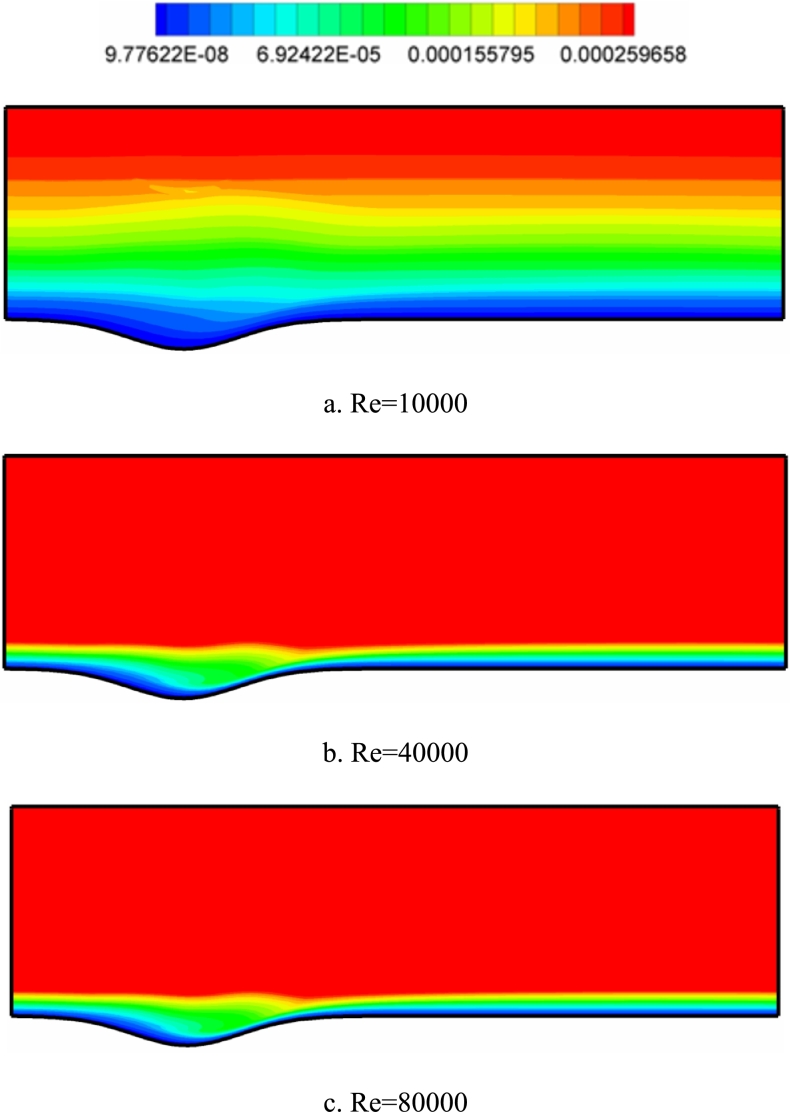
Turbulence viscosity contours (with unit pa.s) in different Reynolds numbers for k-*ω* SST model.

Fig. 16 Turbulence viscosity contours at different Reynolds numbers for the k-*ω* SST model in all contours increases the fluid velocity, enhancing the eddy viscosity. At Re=10000, the velocity of the fluid is reduced due to the influence of the surface friction effects on the areas of the layers above the fluid surface. This reduces the viscosity of the eddy in the area close to the wall surface.

**Figure 17:**
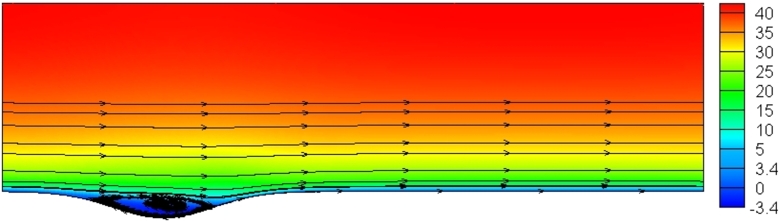
Axial velocity contour (with unit ms^−1^) in Re=10000 for k-*ω* SST model.

Fig. 17 shows the axial velocity contour in the Re=10000 for the k-*ω* SST model. The presence of a wavy area in the channel can affect fluid behavior and affect the stream lines. Due to the deviation of the fluid from the straight line, eddy flows occur in the separation areas. The increment in Reynolds number leads to the production of vortices with higher circulation.

## 6 Conclusion

However, many studies have been performed on laminar and turbulent fluid flows in a variety of geometries, including corrugated geometries [34], [35], [36], [37], [38], [39], [40], [41], [42], in this research, numerical simulation of flow in a two-dimensional corrugated channel was presented in seven different methods of turbulence in two-dimensional space. The results of this study were compared with the results of the DNS method in Re=10000. The results of this study show that in turbulence models that are two-equation. The type of coefficient approximation and the type of flow for which these methods are calculated can have good accuracy or high error in the results presented in each turbulence model. Among the studied cases, the k-*ω* SST method can adequately predict the results of the local friction factor in the flow separation region. Also, the friction factor graph trend is relatively accurate compared to the DNS solution. In Reynolds stress and Spalart–Allmaras methods, estimating the accuracy of friction factor behavior is also acceptable. The presence of a corrugated wall and the friction factor affect the level of flow velocity behavior at different sections and cause a static pressure drop. Turbulence solutions are always associated with high differences. The error in this study compared to the DNS method in calculating some parameters was dependent on the type of turbulence method, estimating the correct shape of the geometry and the number of grids, and the number of time steps of the solution.

## Declarations

### Author contribution statement

Omid Ali Akbari, Davood Toghraie: Conceived and designed the experiments; Performed the experiments; Analyzed and interpreted the data; Contributed reagents, materials, analysis tools or data; Wrote the paper.

Hossein Haghjoo: Conceived and designed the experiments; Analyzed and interpreted the data; Wrote the paper.

Azher M. Abed: Analyzed and interpreted the data.

Mahsa Karimi: Analyzed and interpreted the data; Wrote the paper.

Ali Maghzian: Performed the experiments; Analyzed and interpreted the data; Wrote the paper.

Gholamreza Ahmadi Sheikh Shabani, Amirmasoud Anvari, Nevzat Akkurt: Performed the experiments; Analyzed and interpreted the data; Contributed reagents, materials, analysis tools or data; Wrote the paper.

### Funding statement

This research did not receive any specific grant from funding agencies in the public, commercial, or not-for-profit sectors.

### Data availability statement

No data was used for the research described in the article.

### Declaration of interest's statement

The authors declare no conflict of interest.

### Additional information

No additional information is available for this paper.
